# Examination of sex-specific interactions between gut microbiota and host metabolism after 12-week combined polyphenol supplementation in individuals with overweight or obesity

**DOI:** 10.1080/19490976.2024.2392875

**Published:** 2024-08-25

**Authors:** Kelly M. Jardon, Gijs H. Goossens, Jasper Most, Gianluca Galazzo, Koen Venema, John Penders, Ellen E. Blaak

**Affiliations:** a Department of Human Biology, NUTRIM School of Nutrition and Translational Research in Metabolism, Maastricht University Medical Centre+, Maastricht, The Netherlands; b TiFN, Wageningen, The Netherlands; c Department of Orthopedics, Zuyderland Medical Center, Sittard-Geleen, The Netherlands; d Department of Medical Microbiology, Infectious Diseases and Infection Prevention, NUTRIM School of Nutrition and Translational Research in Metabolism, Maastricht University Medical Centre+, Maastricht, The Netherlands; e Centre for Healthy Eating & Food Innovation, Maastricht University Campus Venlo, Venlo, The Netherlands

**Keywords:** Polyphenols, nutrition, gut microbiota, obesity, sex differences

## Abstract

Polyphenols exert beneficial effects on host metabolism, which may be mediated by the gut microbiota. We investigated sex-specific differences in microbiota composition and interactions with cardiometabolic parameters after polyphenol supplementation in individuals with overweight/obesity. In a double-blind, randomized, placebo-controlled trial, 19 women and 18 men with normal glucose tolerance and body mass index >25 kg/m^2^ received epigallocatechin-3-gallate and resveratrol (EGCG+RES, 282 + 80 mg/d) or placebo supplements for 12 weeks. Fecal microbiota composition (16S rRNA gene amplicon sequencing, V3-V4 region), *in vivo* whole-body fat oxidation (indirect calorimetry), and mitochondrial respiration in permeabilized skeletal muscle fibers (SkM-Ox; *ex vivo* respirometry) were determined pre- and post-intervention. Overall, EGCG+RES supplementation did not affect gut microbiota composition. *Akkermansia, Ruminococcaceae UCG-002, Subdoligranulum*, and *Lachnospiraceae UCG-004* were more abundant, while *Veillonella, Tyzzerella 4, Clostridium innocuum group, Ruminococcus gnavus group, Escherichia-Shigella*, and an uncultured Ruminococcaceae family genus were less abundant in women compared to men. In women, only baseline *Eubacterium ventriosum group* abundance correlated with EGCG+RES-induced changes in SkM-Ox. In men, low *Dorea*, *Barnsiella*, *Anaerotruncus*, *Ruminococcus*, *Subdoligranulum*, *Coprococcus*, *Eubacterium ventriosum group, Ruminococcaceae UCG-003*, and a *Ruminococcaceae* family genus abundance, and high *Blautia* abundance at baseline were associated with improvements in SkM-Ox. Changes in whole-body fat oxidation were not associated with gut microbiota features. We conclude that baseline microbiota composition predicts changes in SkM-Ox as a result of EGCG+RES supplementation in men but not in women. Men may be more prone to diet-induced, gut microbiota-related improvements in cardiometabolic health. These sex-differences should be further investigated in future precision-based intervention studies.

## Introduction

Obesity, type 2 diabetes (T2D) and related cardiometabolic health risks are associated with unfavorable alterations in the gut microbiome.^1,2^ Besides environmental and genetic factors, diet is a key factor in shaping the composition and functionality of the gut microbiota.^1 , 3–5^ Improving cardiometabolic health by modulating the gut microbiota via dietary factors may be effective, but investigating this multifactorial interaction is complex, and responses can be highly individualized.

Both epidemiological and dietary intervention studies suggest that polyphenol-rich diets, such as the Mediterranean diet, are associated with antioxidant, anti-inflammatory and anti-obesity effects, and with a reduced risk of metabolic syndrome and cardiovascular disease in humans.^6,7^ Epigallocatechin-3-gallate (EGCG), a compound naturally present in green tea, and resveratrol (RES), which is highly present in grape skin, are two polyphenols that have the potential to improve glycemic control and reduce inflammation in rodents^8^ and humans.^9–13^ Although health benefits of polyphenols are predominantly attributed to metabolically active peripheral organs, the gut microbiota and related intestinal metabolism might mediate some effects.^14^ After ingestion, polyphenols can accumulate in the large intestine due to poor absorption. There, they are largely metabolized by the intestinal microbiota and broken down into smaller, low molecular-weight bioactive metabolites that can be absorbed and can affect peripheral metabolism.^9,15–18^ Inter-individual differences in microbial capacity to metabolize polyphenols could affect the bioavailability and bioefficacy of polyphenols and their metabolites. Vice versa, polyphenols may modify microbial composition thereby acting as prebiotics and/or may selectively inhibit potential pathogenic species often associated with metabolic disorders.^19,20^ Apart from inter-individual variation in daily intake of polyphenols, inter-individual variation in gut microbiota composition may also determine the susceptibility to polyphenol-induced changes in microbiota structure.^21^

Although polyphenols have been studied extensively, less is known on the direct interaction between polyphenols and the gut microbiota in relation to cardiometabolic health outcomes in humans. In obese mice, a two-month EGCG supplementation affected bile acid metabolism and Verrucomicrobia abundance due to increased *Akkermansia muciniphila* abundance.^7^ The latter has been associated with beneficial metabolic effects in other studies.^22–24^ Additionally, in mice fed a high fat/high sucrose diet, 8-week polyphenol supplementation protected from diet-induced obesity, insulin resistance and intestinal inflammation, which was accompanied by an increased abundance of *Akkermansia spp*.^25^

In humans, we previously reported that 12-week combined RES and EGCG supplementation improved mitochondrial respiration in permeabilized skeletal muscle fibers (SkM-Ox) and postprandial whole-body fat oxidation in individuals living with overweight or obesity.^9^ A subsequent analysis showed that the abundance of the Bacteroidetes phylum, as determined by quantitative PCR, increased in men, but not in women.^10^ In view of the latter interesting but preliminary observations, the present study aimed to investigate sex differences in the effects of 12-week EGCG+RES supplementation on gut microbiota composition in more detail, using 16S rRNA gene amplicon sequencing. Additionally, we assessed whether baseline microbial composition was predictive for EGCG+RES-induced changes in whole-body fat oxidation and SkM-Ox in women and men.

## Materials and methods

### Study design and population

This study was a secondary analysis of a larger randomized, double-blind, placebo-controlled, parallel-designed study, aiming to investigate the effects of combined EGCG+RES supplementation on insulin sensitivity, substrate oxidation and SkM-Ox, performed at the Maastricht University Medical Center+ (MUMC+) in Maastricht, the Netherlands.^9,10^ The study population consisted of healthy Caucasian men (*n* = 21) and women (*n* = 21) aged between 20 and 50 y old and living with overweight or obesity body mass index (BMI > 25 kg/m^2^). Participants had to be weight stable (<2 kg body weight change within 3 months before inclusion), untrained (<3 h organized sports activities per week), were normal glucose tolerant and had a normal blood pressure. Exclusion criteria were the use of any antibiotics or medication/supplements that may interfere with insulin sensitivity and substrate metabolism for 3 months before entering the study and having a daily intake of caffeine (>600 mg), green tea (>3 cups) and alcohol (>20 g). Individuals with a vegetarian or other special diet were excluded from participation. Detailed inclusion and exclusion criteria for study participants were published before.^9^ The subjects gave written informed consent for participation in this study. The protocol was approved by the Medical Ethics Committee of the MUMC+ (NL31421.068.10) and registered at ClinicalTrials.gov (identifier NCT02381145). All procedures were performed according to the Declaration of Helsinki (October 2008).

Briefly, sample collection and clinical measurements, as described below, were performed during the clinical investigation days before and in the last week of supplementation.^9^ Subjects were instructed to maintain their habitual lifestyle pattern throughout the study. Regular control visits (weeks 2, 4 and 8) and assessments of dietary intake (3-day food records, week 0, 4 and 12) and physical activity were scheduled throughout the study.

### Supplements

The supplements were commercially available and provided by Pure Encapsulations Inc. (Sudbury, MA, USA). All capsules were manufactured, tested and checked in accordance with standards of the European Union’s Good Manufacturing Practices (GMP) requirements. EGCG capsules contained 94% EGCG (141 mg per capsule) and RES capsules 20% trans-resveratrol (40 mg per capsule).^9^ Two kinds of PLA capsules (microcrystalline cellulose) were used for blinding. One capsule of each supplement (EGCG and resveratrol or both blinded placebo capsules) was ingested during breakfast and dinner. This protocol was maintained until the last measurement. On clinical investigation days (CIDs), subjects ingested the capsules before arrival at the university (between 07:00 and 08:00 h). After completion of the study, returned capsules were counted for compliance.

### Fecal sampling and gut microbiota composition

Fecal samples were collected at home and stored in the participants’ freezer at −20°C for a maximum of 24 h before handing over to the researchers. On arrival during the CIDs, the samples were stored at −80°C upon analysis. Samples were kept frozen during transport from collection to storage and from storage to analysis. Sequencing of the V3-V4 region of the 16S rRNA gene was performed to determine microbiota composition as described previously.^26,27^ In short, QIAamp Fast DNA Stool Mini Kits (Qiagen, Venlo, the Netherlands) were used for genomic DNA isolation. Barcoded amplicons from the V3-V4 region of 16S rRNA genes were generated using a 2-step PCR. In the first step, 10–25 ng genomic DNA was used as a template for the first PCR with a total volume of 50 μl using the 341F (5’-CCTACGGGNGGCWGCAG-3’) and 785 R (5’-GACTACHVGGGTATCTAATCC-3’) primers appended with Illumina adaptor sequences. PCR products were purified (QIAquick PCR Purification Kit), and the size of the PCR products was checked on a Fragment analyzer (Advanced Analytical, Ankeny, US) and quantified by fluorometric analysis (Qubit™ dsDNA HS Assay Kit). Purified PCR products were used for the second PCR in combination with sample-specific barcoded primers (Nextera XT index kit, Illumina, San Diego, CA, USA). Subsequently, PCR products were purified, checked on a Fragment analyzer and quantified, followed by equimolar multiplexing and sequencing on an Illumina MiSeq with the paired-end (2×) 300 bp protocol (Illumina, Eindhoven, The Netherlands). The sequencing run was analyzed with the Illumina CASAVA pipeline (v1.8.3) with demultiplexing based on sample-specific barcodes. Quantitative Insights Into Microbial Ecology 2 (QIIME2) software was used for initial microbial analyses.^28^ Reads were imported and quality filtered and dereplicated with the q2-data2 plugin. Subsequently, the dada2 plugin was used with paired-end reads with truncation of the primer sequences and trimming of the reads. The resulting data were used in the q2-phylogeny plugin to generate a tree for phylogenetic diversity analyses. The sequences were taxonomically classified using Silva (version 132) as a reference 16S rRNA gene database.

### Whole-body fat oxidation and ex vivo mitochondrial respiration in permeabilized skeletal muscle fibers

As described elsewhere, 12-week EGCG+RES supplementation significantly increased SkM-Ox and stimulated whole-body fat oxidation compared to placebo.^9^ Additionally, in a preliminary analysis, sex-specific interactions between EGCG+RES intake, fat oxidation and the gut microbiota were found.^10^ Therefore, we only included SkM-Ox and whole-body fat oxidation as metabolic parameters in the present analyses. A high-fat mixed-meal (HFMM) test (2.6 MJ, 61.2 energy% fat) was performed to assess whole-body fat oxidation before (*t* = 0 min) and for 4 h after ingestion of the HFMM by means of indirect calorimetry, using an open-circuit ventilated hood system (Omnical, Maastricht University, Maastricht, The Netherlands). Skeletal muscle mitochondrial oxidative capacity was measured by *ex vivo* high-resolution respirometry (Oroboros Instruments, Innsbruck, Austria) using permeabilized skeletal muscle fibers that were isolated from biopsies (*m. vastus lateralis)*, which were collected after an overnight fast under local anesthesia. Full procedures have been described elsewhere.^29^

### Statistical analysis

#### Clinical parameters

Normality of clinical data was assessed using Shapiro–Wilk testing and visual inspection where necessary. Differences in participants’ characteristics at week 0 were tested by independent samples t-test for normally distributed data and a Mann–Whitney test for non-normal distributed data. Intervention effects were analyzed by using a repeated-measures ANOVA, with time and treatment as factors. In case of a significant time × treatment interaction, we performed post hoc analyses with Bonferroni correction to determine within-group effects. Clinical data are expressed as mean ± SD where possible, with a significance level of *p* < .05. Analyses were performed in IBM SPSS Statistics 28.

#### Gut microbiota

Sex differences in relative abundance of individual microbial taxa and alpha-diversity were assessed using non-parametric Mann–Whitney testing. To assess intervention effects on individual microbial taxa, generalized linear mixed models with negative binomial distribution were used. Analyses were performed in R with the glmmADMB package.^30^ Genera with more than 70% zero values were filtered out of the model. Accordingly, the model was adjusted to sex (ANOVA). To correct for multiple comparisons and limit false positive outcomes, P-values were corrected for False Discovery Rate (FDR) using the Benjamini–Hochberg method. Due to the exploratory nature of this study, FDR-adjusted P-values (Q-values) with a significance set at Q < 0.2 were used to describe the data. Microbial community profile analyses (Bray-Curtis and Jaccard) for assessing both overall sex-differences and intervention effects within the EGCG+RES or PLA group were performed with PERMANOVA. Microbial diversity analyses (observed richness and Shannon index) were performed by Mann–Whitney (sex-differences) or Wilcoxon rank-sum testing (intervention effects) using Microbiome Analyst software.^31^ Here, the significance was set at *p* < .05. Spearman correlation analysis was performed to identify correlations between pre-intervention and intervention induced changes in individual microbial taxa and changes in metabolic outcome parameters (Q < 0.2).

## Results

### Participant inclusion and characteristics

**Figure 1.**
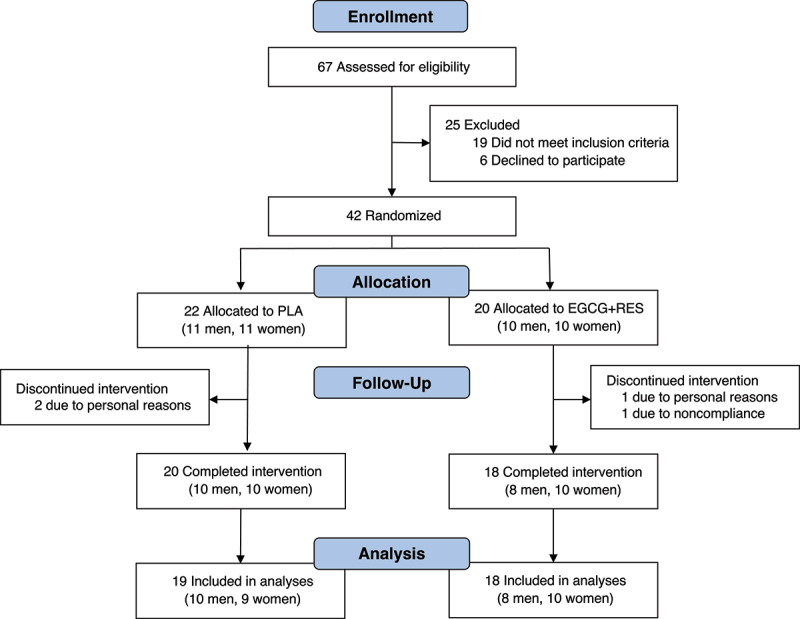
Flowchart of participant enrolment and eligibility.

Between August 2012 and December 2013, 67 men and women with overweight or obesity and normal glucose tolerance were screened for eligibility (Figure 1). Forty-two individuals were randomized to either the EGCG+RES (*n* = 20) or placebo (*n* = 22) study arm. In total, four individuals (three men, one woman) discontinued the intervention due to personal circumstances (*n* = 3) or noncompliance with respect to supplementation (*n* = 1). Additionally, data of one individual in the PLA group were excluded from the analysis due to reported diarrhea. In total, data of 37 individuals were included in the analysis and are reported here.

EGCG+RES, epigallocatechin-3-gallate + resveratrol; PLA, placebo.

Clinical characteristics of the 37 individuals who completed the study are summarized in Supplemental Table S1, as also previously reported.^10^ Men and women were equally distributed both in the total study population and within the PLA and EGCG+RES groups. There were no differences between the EGCG+RES and PLA groups with respect to general clinical characteristics. Overall, women had a lower body weight, waist circumference, waist/hip ratio and visceral fat mass, while their body fat percentage was higher (all *p* < .05). Although still within the criteria for normal glucose tolerance, fasting glucose was higher in men (5.29 ± 0.23 mmol/l) compared to women (5.02 ± 0.44 mmol/l, *p* < .01). Additionally, men had higher concentrations of the plasma inflammatory cytokines interleukin-8 and tumor necrosis factor alpha (TNF-α, *p* < .05), and a lower plasma HDL concentration (*p* < .001). Men had a higher total daily energy intake compared to women (*p* = .010), but percentages of macronutrient intake, fiber consumption and alcohol intake were similar (Supplemental Table S1). Habitual dietary intake was similar in the EGCG+RES and PLA groups.^9^ No sex-specific pre-intervention differences in skeletal muscle oxidative capacity and substrate oxidation were detected, as reported previously.^10^

### EGCG+RES supplementation stimulated whole-body fat oxidation and SkM-Ox

As published,^9^ SkM-Ox increased after 12-week EGCG+RES supplementation as compared to PLA (Supplemental Table S2). In line, fasting and postprandial whole-body fat oxidation was preserved from decline in the EGCG+RES group as compared to placebo, which was preserved throughout (fasting fat oxidation: PLA −14.3%, EGCG+RES +10.5%; postprandial fat oxidation: −16.5%, EGCG+RES +8.4%). There were no sex-specific differences in the EGCG+RES-induced changes in both SkM-Ox and whole-body fat oxidation.

### Gut microbiota composition and diversity was not altered after 12-week EGCG+RES supplementation

**Figure 2.**
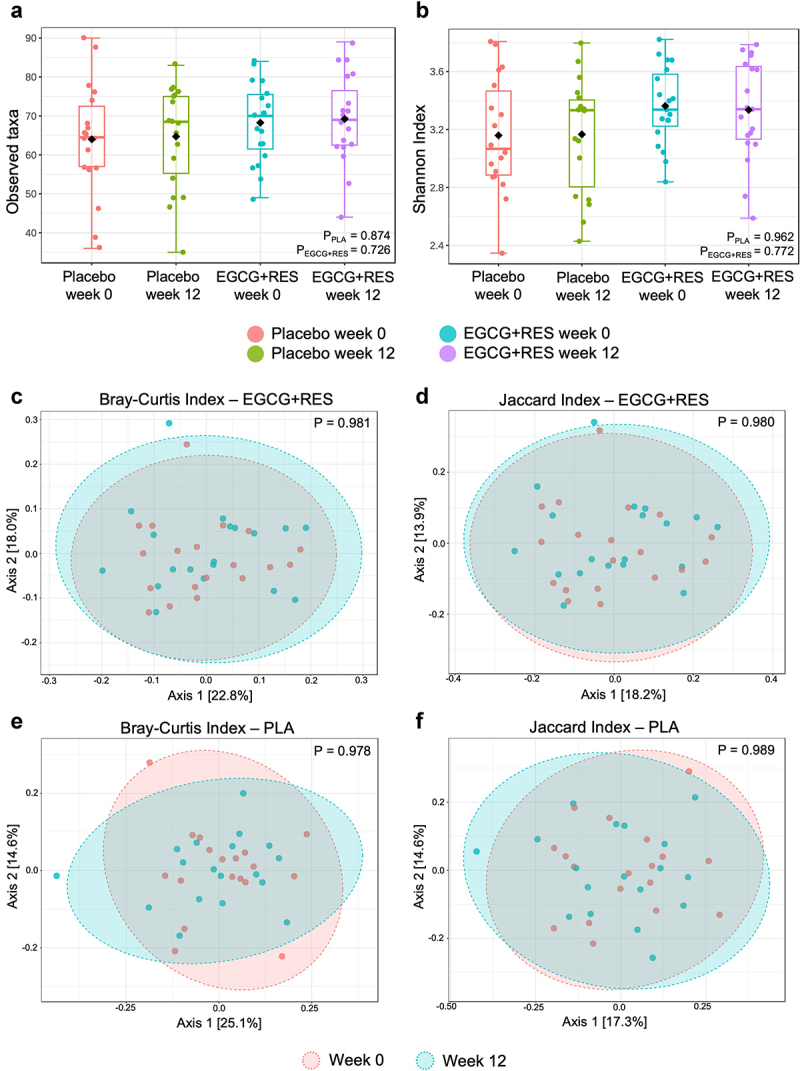
Effects of 12-weeks EGCG+RES supplementation on measures of fecal microbiota diversity and community structure. (a) Observed taxa as indicator of microbial richness, and (b) microbial diversity as assessed by the Shannon diversity index at baseline (week 0) and after the 12-week intervention. Boxplots indicate the median and interquartile ranges (IQR). (c-f) PCoA plots using Bray-Curtis dissimilarity and Jaccard indices as indicators of overall microbiota community shifts within the EGCG+RES group (c-d), and within the PLA group respectively (e-f). *p* < .05 was considered as statistically significant. EGCG+RES, epigallocatechin-3-gallate and resveratrol.

Supplementation with EGCG+RES did not induce changes in the relative abundance of individual microbial taxa at genus level, adjusted for sex (Supplemental Table S3). In line, microbial richness (observed taxa; EGCG+RES: p = 0.726, PLA: p = 0.874) and diversity (Shannon Index; EGCG+RES: p = 0.773, PLA: p = 0.962) did not change after intervention (Figure 2a–b). No dissimilarities in the overall microbial community structure were found between week 0 and week 12, both within the EGCG+RES and placebo group (Bray-Curtis Index: EGCG+RES: p = 0.981, PLA: p = 0.980; Jaccard Index: EGCG+RES: p = 0.978, PLA: p = 0.989) (Figure 2c–f).

### Sex differences in gut microbiota composition and community structure but not in richness and diversity

**Figure 3.**
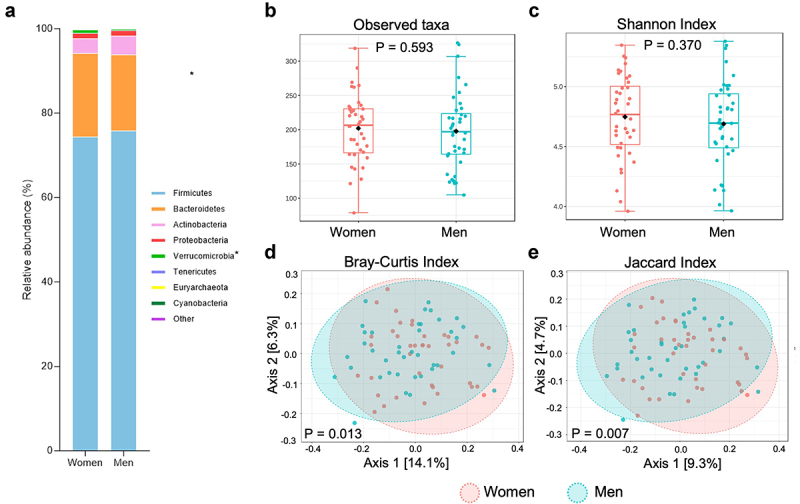
Overall comparison of gut microbial profiles of men versus women. (a) Relative abundance of microbial taxa on phylum level in men versus women. FDR adjusted P-values of Q < 0.2, (*) were considered to be statistically significant. (b) Total observed ASVs (B) and Shannon index (c) as a measure for alpha diversity. Boxplots indicate the median and interquartile ranges (IQR). PCoA plots using the Bray-Curtis index (d) and Jaccard index to visualize the overall microbiota community variation. For (b-e): statistical significance (*p* < .05).

In the total population, gut microbiota composition showed differences between men and women. At phylum level, the relative abundance of Verrucomicrobia was higher in women, which remained statistically significant after FDR correction (p = 0.003, Q = 0.022) (Figure 3a). At genus level, relative abundances of the potent short-chain fatty acid (SCFA) producers *Akkermansia* (p = 0.003, Q = 0.103), *Ruminococcaceae UCG-002* (p = 0.007, Q = 0.124), *Subdoligranulum* (p = 0.007, Q = 0.124), and *Lachnospiraceae UCG-004* (p = 0.010, Q = 0.129) were higher in women when combining pre- and post-intervention data, while the relative abundances of *Veillonella* (p = 0.006, Q = 0.124), *Tyzzerella 4* (p = 0.00, Q = 0.088), *Clostridium innocuum group* (p = 0.001, Q = 0.088), *Ruminococcus gnavus group* (p = 0.003, Q = 0.103), *Escherichia-Shigella* (p = 0.006, Q = 0.124), and an uncultured genus of the Ruminococcaceae family (p = 0.008, Q = 0.124) were higher in men (Table 1). There were no significant sex differences in the number of observed taxa (p = 0.593) and Shannon Index (p = 0.370), reflecting alpha-diversity (Figure 3b,c). There was a sex-specific clustering of microbial communities, as indicated by the Bray-Curtis (p = 0.013) and Jaccard index (p = 0.007) (Figure 3d,e).

**Table 1.** Overall differential abundant taxa on genus level of women versus men.

Genus *Relative abundance, %*	Women	Men	P-value	Q-value
	n = 19	n = 18		
*Tyzzerella 4*	0.02 ± 0.07	0.05 ± 0.07	0.001	0.088*
*Clostridium innocuum group*	0.39 ± 0.28	0.52 ± 0.26	0.001	0.088*
*Ruminococcus gnavus group*	0.39 ± 0.42	0.69 ± 0.65	0.003	0.103*
*Akkermansia*	0.90 ± 1.35	0.32 ± 0.74	0.003	0.103*
*Ruminococcaceae UCG-002*	2.25 ± 2.33	1.23 ± 1.88	0.007	0.124*
*Subdoligranulum*	1.28 ± 1.25	0.65 ± 0.74	0.007	0.124*
*Veillonella*	0.09 ± 0.13	0.14 ± 0.12	0.006	0.124*
*Escherichia-Shigella*	0.51 ± 0.46	0.77 ± 0.73	0.006	0.124*
*Ruminococcaceae uncultured genus*	0.56 ± 0.62	0.6 ± 0.63	0.008	0.124*
*Lachnospiraceae UCG-004*	0.11 ± 0.20	0.04 ± 0.16	0.010	0.129*

Data are presented as mean ± SD relative abundance (%), significance based on Mann-Whitney U testing. *Indicates a significant difference in women and men (Q < 0.2 (= False Discovery Rate adjusted P-value)). The women versus men comparison is based on combined pre- and post-intervention data.

#### Pre-intervention gut microbiota composition is associated with egcg+res-induced changes in SkM-Ox in men

**Figure 4.**
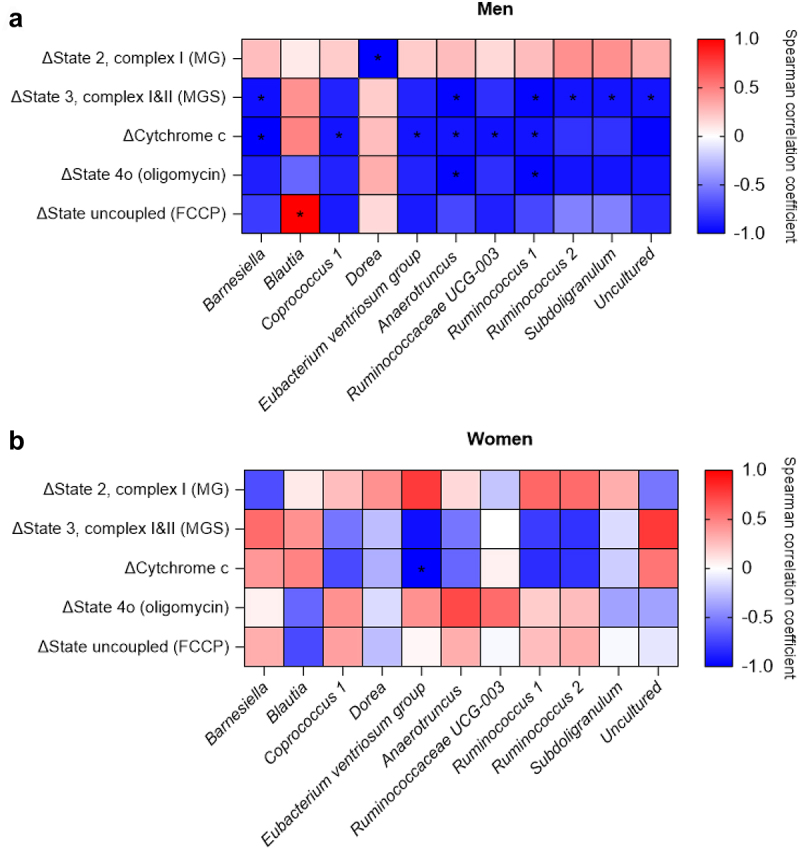
Spearman’s correlation heatmaps of pre-intervention microbiota composition and changes in markers of SkM-ox. Significant correlations in men (a) and in women (b) as results of EGCG+RES supplementation. Data on the x-axis are represented as changes in SkM-ox (week 12-week 0, δpmol O₂/mg muscle), after additions of malate+glutamate (MG, adp-stimulated state 2, complex I), malate+glutamate+succinate (MGS, state 3, complex I&II), cytochrome c (mitochondrial integrity), oligomycin (mitochondrial proton leak), and carbonyl cyanide-4-phenylhydrazone (FCCP, maximal mitochondrial respiration). A red color indicates positive correlations and blue negative. A ‘*’ indicates correlations with an fdr-adjusted P-value of Q < 0.2. Uncultured, uncultured bacterium of the Ruminococcaceae family.

We next investigated whether EGCG+RES-induced improvements in SkM-Ox and fat oxidation are determined by pre-intervention gut microbiota composition. Indeed, we found that the baseline abundance of specific genera is correlated with the EGCG+RES-induced changes in SkM-Ox, but these associations were mainly found in men (Figure 4, Supplemental Table S4). More specifically, in men, baseline *Dorea* abundance was negatively associated with changes in state 2, ADP-stimulated complex I-linked respiration upon addition of malate + glutamate (Figure 4a). Baseline abundances of *Barnsiella, Ruminococcus 1, Ruminococcus*, *Subdoligranulum* and an uncultured bacterium of the *Ruminococcaceae* family were negatively correlated with changes in state 3, complex I and II-linked respiration, assessed by addition of succinate after malate + glutamate. Baseline abundances of *Barnsiella*, an uncultured bacterium of the *Ruminococcaceae* family, *Coprococcus 1*, *Eubacterium ventriosum group*, *Anaerotruncus*, *Ruminococcaceae UCG-003* and *Ruminococcus 1* were negatively associated with changes in respiration after introducing cytochrome c. Baseline *Anaerotruncus* and *Ruminococcus 1* abundance were negatively correlated with changes in state 4o-linked respiration. Lastly, in men, a positive correlation was found between baseline *Blautia* abundance and changes in maximal mitochondrial respiration (state uncoupled (FCCP)). In women, a negative correlation was found between baseline *Eubacterium ventriosum group* abundance and changes in cytochrome c induced respiration (Figure 4b). There were no significant correlations between the baseline abundance of specific genera and EGCG+RES-induced changes in whole-body fasting and postprandial fat oxidation (Supplemental Table S5).

### Changes in microbial taxa relative abundance correlate with an EGCG+RES induced increase SkM-Ox

**Figure 5.**
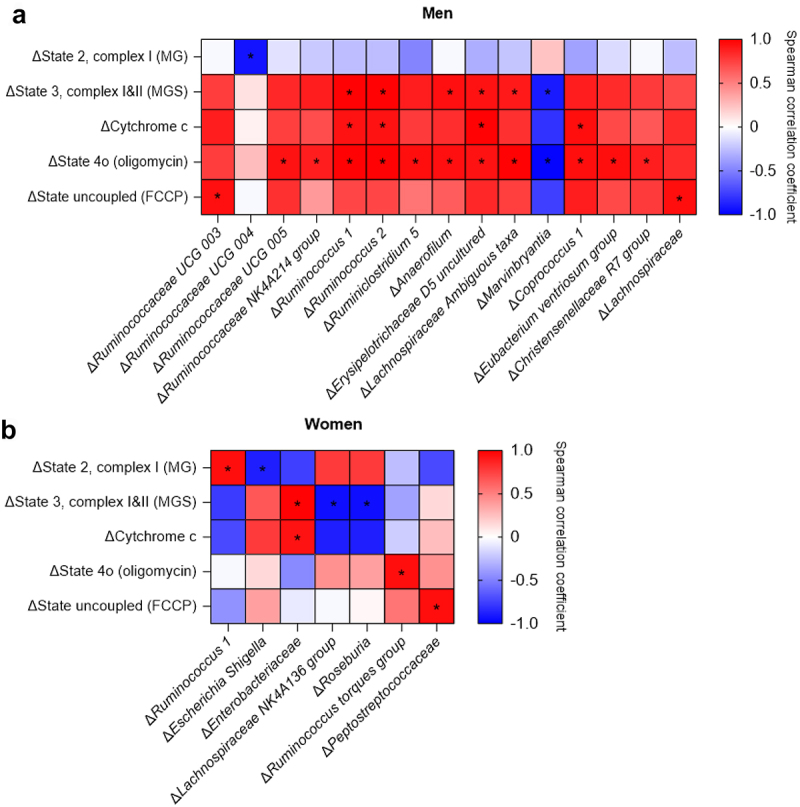
Spearman’s correlation heatmaps of EGCG+RES induced alterations in individual microbial taxa and changes in markers of SkM-ox. Significant correlations in men (a) and in women (b) as results of EGCG+RES supplementation. Data on the x-axis are represented as changes in SkM-ox (week 12-week 0, δpmol O₂/mg muscle), after additions of malate+glutamate (MG, adp-stimulated state 2, complex I), malate+glutamate+succinate (MGS, state 3, complex I+II), cytochrome c (mitochondrial integrity), oligomycin (mitochondrial proton leak), and carbonyl cyanide-4-phenylhydrazone (FCCP, maximal mitochondrial respiration). A red color indicates positive correlations and blue negative. A ‘ * ’ indicates correlations with an fdr-adjusted P-value of Q < 0.2.

To further explore the relationship between the gut microbiota and changes in metabolic outcomes, we investigated whether the EGCG+RES-induced increase in SkM-Ox is related to shifts in individual microbial taxa. In men, improvements in SkM-Ox are related to an increase in several genera with SCFA producing potential, as shown by the merely positive correlations in Figure 5a and Supplemental Table S6. However, a shift in *Ruminococcaceae UCG 004* abundance was negatively associated with changes in state 2, ADP-stimulated complex I-linked respiration. Changes in abundances of five individual genera were positively correlated, and one genus was negatively correlated with changes in state 3, complex I and II-linked respiration. Shifts in abundances of four individual genera also showed a positive correlation with changes in respiration after introducing cytochrome c. Additional positive correlations were found between changes in state 4o-linked respiration and shifts in 11 individual microbial genera, while 1 was negatively correlated. Lastly, in men, shifts in two individual genera showed a positive correlation with changes in maximal mitochondrial respiration.

In women, correlations between EGCG+RES-induced shift in microbiota composition and SkM-Ox were less pronounced and less related to an increase in SCFA producing bacteria (Figure 5b, Supplemental Table S7). Here, shifts in one individual genus had a positive and one genus had a negative correlation with state 2, ADP-stimulated complex I-linked respiration (Figure 5b). EGCG+RES-induced shifts in the relative abundance of one genus were positively and two were negatively correlated with changes in state 3, complex I and II-linked respiration. Changes in abundance of one genus showed a positive correlation with changes in respiration after introducing cytochrome c, and two genera with maximal respiration in women.

## Discussion

Preliminary findings indicated that favorable polyphenol-induced effects on human fat metabolism may be linked to gut microbiota composition, specifically in men.^10^ In the current study, we therefore performed more comprehensive gut microbiota analyses using 16S rRNA sequencing to investigate whether microbial composition associates with EGCG+RES-induced improvement in whole-body fat oxidation and SkM-Ox in women and men with overweight or obesity. Firstly, we reported that 12-week EGCG+RES supplementation did not alter gut microbiota composition. Overall, we found distinct, sex-specific differences in microbial community structure and higher relative abundances of potent SCFA-producing bacteria in women. Interestingly, our findings also confirm that pre-intervention microbiota composition may be predictive for polyphenol-induced changes in SkM-Ox in men but not in women. In line, changes in SkM-Ox were more abundantly correlated with shifts in individual microbial taxa in men versus women. Overall, it seems that the gut microbiota may be a more relevant mediator of polyphenol-induced changes in skeletal oxidative capacity in men and not in women.

Although ECCG+RES supplementation did not alter characteristics of the gut microbiota in the current study at group level, results indicate that baseline (week 0) microbial profiles as well as changes therein may predict polyphenol-induced changes in SkM-Ox in men but not in women. In men, bacteria present at baseline related to the *Ruminococcus* genus, also known as SCFA producers,^32^ were negatively correlated with the increase in SkM-Ox. Additionally, we found a positive correlation between EGCG+RES-induced changes in *Ruminococcus* and increased SkM-Ox. Likewise, the SCFA producing genera related to *Lachnospiraceae* were less abundantly present in men compared to women, but the EGCG+RES-induced increase in their abundance was associated with improved SkM-Ox. *Subdoligranulum*, known for its ability to produce butyrate,^33^ also had a lower presence in men and was negatively associated with changes in SkM-Ox. The finding that microbial-derived SCFA may affect oxidative capacity or fat oxidation is in line with previous studies of our group, showing that distal colonic SCFA administration led to a pronounced dose-dependent increase in fasting fat oxidation in healthy males with overweight. Of note, the changes in EGCG and resveratrol metabolites did not differ between males and females. Additionally, there were no significant correlations between the differential taxa between males and females and plasma concentrations of EGCG or resveratrol metabolites. Hence, our findings suggest that the sex-specific interactions between microbiota and host metabolism are not explained by differences in circulating polyphenol metabolite availability. Taken together, this consistently implies that a low abundance of SCFA producers at baseline may be a good predictor of polyphenol-induced changes due to the higher window for improvement in men but to a lesser extent in women. The predictive capacity of the baseline microbial profile for metabolic outcomes is in line with the relationship between gut microbial composition and acute glycemic or postprandial response after food intake^34,35^ and longer-term dietary interventions.^36^ Our data expand these findings by showing the sex-specific nature of this relationship during polyphenol intervention. This further supports our hypothesis that effects of polyphenol interventions depend on an individual’s microbial profile.

Overall, women seem to have higher relative abundances of microbial taxa that have been linked with beneficial health outcomes.^37^ In line, bacteria of the *Akkermansia* genus, the main taxon of the Verrucomicrobia phylum, here higher in women, has been associated with favorable metabolic traits, including a reduced risk of obesity, type 2 diabetes and nonalcoholic fatty liver disease.^22,38^ These protective effects may be linked to the ability to produce active gut metabolites such as SCFA that, in turn, can stimulate glucagon-like protein 1 (GLP-1) secretion and regulate inflammatory responses.^33,39^ We found that the genera *Veillonella, Tyzzerella 4, Clostridium innocuum group, Ruminococcus gnavus group, Escherichia-Shigella*, and an uncultured genus of the Ruminococcaceae family were more abundantly present in men. Both *Tyzzerella 4* and the *Ruminococcus gnavus group* have been linked to an augmented inflammatory status.^40,41^ *Escherichia-Shigella, Clostridium innocuum group* and *Veillonella* are not strongly linked to cardiometabolic health. In contrast to the microbial genera with a higher presence in women, these genera are mainly related to neutral or detrimental health effects.^42–44^ In this study, microbial richness and diversity were not different between men and women, but we did find sex-specific differences in specific bacterial taxa as well as a distinct bacterial community structure. In line, human studies investigating sex-specific differences in gut microbial characteristics reported overall community differences between men and women, whilst α-diversity was higher in women compared to men.^45,46^ Findings regarding sex differences in specific microbial taxa are more inconsistent in literature and depend on the study population.^46^ Taken together, the included women in the current cohort seemed to have more favorable gut microbiota characteristics compared to men. Sex hormones may be a key determinant of these sex-differences^47^ but also external factors, including lifestyle choices like diet^1,3^ and gastrointestinal transit time^48^ are significant determinants of the composition and diversity of the gut microbiota.^49^ In the current study, the reported habitual dietary intake was not different between women and men,^50^ however other factors were not determined.

A strength of the present study is that individuals were metabolically phenotyped in detail with respect to substrate metabolism. Furthermore, in contrast to our previous study in which we performed qPCR analysis to provide absolute quantitative information on only a limited, pre-selected subset of microbes,^10^ we now applied 16S rRNA gene sequencing to generate extensive information on the gut microbiota composition, allowing more detailed insights into sex-specific interactions between gut microbiota composition and host metabolism. However, this study also has some limitations. First, we did not include assessments of functional markers of gut microbiota activity, including circulating and fecal concentrations of gut metabolites, including SCFA, which would have provided more insight into the polyphenol-gut microbiota-peripheral metabolism crosstalk.^51,52^ Secondly, metagenomic analyses would have provided an even more detailed analysis of microbiomefunctionality in relation to polyphenol intake and cardiometabolic health.^53^ Additionally, other factors affecting gut health, including gastrointestinal transit time, an important determinant of the production of gut metabolites and metabolic health, may be taken into account in future studies.^54^ The gastrointestinal transit time may be different in women versus men,^48^ which may also be linked to the observed sex-differences in gut microbiota composition in this study. Lastly, our findings cannot exclude that other sex-specific differences in physiology, including sex hormones and their effects on human metabolism, play a role EGCG+RES induced effects on SkM-Ox. Although this study cannot confirm a direct causal role of the gut microbiota in this relation, the sex-specific correlations should be investigated further in future studies.

To conclude, combined EGCG+RES supplementation did not induce changes in the gut microbiota of men and women with normal glucose tolerance and overweight or obesity. Importantly, microbiota composition seems to be predictive for polyphenol-induced changes in SkM-Ox in men but not in women.^9^ This is related to a lower abundance of potent SCFA producers in men specifically. These data thus indicate a sex-specific relationship between the microbiome and metabolic health. Thus far, sex has been largely underestimated as determinant of the interaction between an individual’s gut microbiota profile and response to dietary or therapeutic interventions. Based on our findings and in line with previous literature, we suggest that future studies investigating the interaction between gut microbiota and host metabolism in humans should consider subgroup-specific analyses, taking sex, metabolic and microbial phenotypes into account. With a better understanding of the complex interactions between these factors, a more personalized interventions to improve metabolic health and prevent the development of chronic diseases can be established.

## Supplementary Material

Supplemental Material

## Data Availability

The published article and supplemental information contain the clinical data used to generate the figures in the paper. Data generated by 16S rRNA sequencing and corresponding metadata are deposited in the National Center for Biotechnology Information Sequence Read Archive (NCBI SRA). Any other information required to reanalyze the data reported in this paper is available from the lead contact upon reasonable request.

## List of abbreviations

ASV: amplicon sequence variant
BMI: body mass index
BP: blood pressure
CHO: carbohydrates
CID: clinical investigation day
EGCG: epigallocatechin-3-gallate
FDR: false discovery rate
G: glutamate
GLP-1: glucagon-like peptide 1
GMP: good manufacturing practices
HbA1c: glycated hemoglobin
HDL: high density lipoprotein
HFMM: high-fat mixed-meal
HOMA-IR: homeostasis model assessment of insulin resistance
IR: insulin resistance
M: malate
MJ: megajoule
NGT: normal glucose tolerance
PCoA: Principal Coordinates Analysis
PLA: placebo
RES: resveratrol
SCFA: short-chain fatty acid
SkM-Ox: mitochondrial respiration in permeabilized skeletal muscle fibers
S: succinate
T2D: type 2 diabetes
TNF-α: tumor necrosis factor-α
VAT: visceral adipose tissue
