# Supplementary material for: Examination of sex-specific interactions between gut microbiota and host metabolism after 12-week combined polyphenol supplementation in individuals with overweight or obesity
Source: Gut Microbes. 2024 Aug 25;16(1):2392875. doi: 10.1080/19490976.2024.2392875 (PMC11346568; doi:10.1080/19490976.2024.2392875)
Supplement: Supplemental Material [file KGMI_A_2392875_SM2205.zip › Revised_Manuscript_clean.docx]

**Supplemental information**

**Supplemental Table 1.** Clinical characteristics of participants stratified by sex and treatment at week 0.

|  | **Sex** | | | **Treatment** | | |
| --- | --- | --- | --- | --- | --- | --- |
|  | **Women**  **(n = 19)** | **Men**  **(n = 18)** | **P-value**  **(sex)** | **PLA**  **(n = 19)** | **EGCG+RES**  **(n = 18)** | **P-value (group)** |
| Women, n (%) |  |  |  | 9 (47) | 10 (55) | 0.121 |
| Age (years) | 35.6 ± 10.6 | 40.2 ± 8.9 | 0.170 | 39.5 ± 10.0 | 36.1 ± 9.9 | 0.296 |
| Systolic BP (mm Hg) | 113 ± 11 | 118 ± 10 | 0.152 | 114 ± 12 | 117 ± 8 | 0.296 |
| Diastolic BP (mm Hg) | 75 ± 10 | 77 ± 8 | 0.587 | 77 ± 12 | 76 ± 8 | 0.833 |
| *Anthropometrics and body composition* |  |  |  |  |  |  |
| Weight (kg) | 83.2 ± 9.5 | 96.3 ± 14.8 | 0.004* | 87.1 ± 12.5 | 92.2 ± 15.0 | 0.275 |
| BMI (kg/m^2^) | 29.2 ± 2.5 | 30.0 ± 3.5 | 0.460 | 29.3 ± 3.3 | 29.9 ± 2.6 | 0.504 |
| Waist circumference (cm) | 89.5 ± 8.3 | 99.5 ± 10.3 | 0.003* | 92.9 ± 9.8 | 95.9 ± 11.3 | 0.395 |
| Waist-to-hip ratio | 0.80 ± 0.07 | 0.94 ± 0.08 | <0.001* | 0.88 ± 0.87 | 0.87 ± 0.10 | 0.658 |
| Body fat (%) | 36.6 ± 3.7 | 24.4 ± 4.0 | <0.001* | 29.7 ± 8.4 | 31.6 ± 6.0 | 0.453 |
| Visceral fat (g) | 397.7 ± 150.0 | 544.7 ± 222.4 | 0.024* | 488.4 ± 197.3 | 448.9 ± 207.3 | 0.556 |
| *Glucose metabolism* |  |  |  |  |  |  |
| Fasting glucose (mmol/l) | 5.02 ± 0.44 | 5.29 ± .23 | 0.026* | 5.11 ± 0.36 | 5.19 ± 0.39 | 0.522 |
| 2-hr glucose (mmol/l) | 5.48 ± 1.08 | 5.27 ± 1.01 | 0.546 | 5.41 ± 1.02 | 5.34 ± 1.08 | 0.842 |
| HbA1c (%) | 5.14 ± 0.26 | 5.16 ± 0.29 | 0.787 | 5.17 ± 0.27 | 5.12 ± 0.27 | 0.566 |
| *Blood metabolites* |  |  |  |  |  |  |
| IL-6 (pg/ml) | 0.88 ± 0.57 | 0.79 ± 0.58 | 0.632 | 0.89 ± 0.73 | 0.77 ± 0.31 | 0.537 |
| IL-8 (pg/ml) | 8.13 ± 3.08 | 10.85 ± 3.11 | 0.011* | 9.13 ± 2.76 | 9.80 ± 3.93 | 0.550 |
| TNF-a (pg/ml) | 2.33 ± 0.37 | 3.30 ± 0.92 | <0.001* | 2.88 ± 0.95 | 2.69 ± 0.74 | 0.493 |
| Serum total cholesterol (mmol/l) | 6.06 ± 1.42 | 5.90 ± 0.86 | 0.674 | 5.92 ± 1.29 | 6.04 ± 1.05 | 0.771 |
| Serum HDL (mmol/l) | 1.44 ± 0.31 | 1.03 ± 0.25 | <0.001* | 1.22 ± 0.34 | 1.27 ± 0.36 | 0.675 |
| Serum LDL (mmol/l) | 4.41 ± 1.35 | 4.49 ± 0.77 | 0.814 | 4.44 ± 1.23 | 4.45 ± 0.95 | 0.983 |
| *Habitual dietary intake* |  |  |  |  |  |  |
| Energy intake (MJ/day) | 7.3 ± 2.0 | 9.8 ± 3.0 | 0.010* | 8.3 ± 2.5 | 8.8 ± 3.3 | 0.646 |
| Habitual fat intake (en%) | 36.0 ± 5.3 | 38.7 ± 7.1 | 0.226 | 37.8 ± 6.5 | 36.7 ± 6.2 | 0.602 |
| Habitual protein intake (en%) | 15.5 ± 3.4 | 15.3 ± 3.2 | 0.981 | 16.1 ± 3.3 | 14.5 ± 3.0 | 0.158 |
| Habitual CHO intake (en%) | 46.5 ± 5.7 | 43.9 ± 6.6 | 0.239 | 45.4 ± 7.1 | 45.0 ± 5.0 | 0.852 |
| Habitual fiber intake (g/MJ) | 1.7 ± 0.7 | 1.8 ± 0.5 | 0.935 | 1.9 ± 0.6 | 1.6 ± 0.6 | 0.097 |
| Habitual alcohol consumption (g) | 5.3 ± 10.5 | 7.8 ± 10.3 | 0.363 | 3.7 ± 8.3 | 9.8 ± 11.7 | 0.290 |

Values represented as mean ± SD. Pre-intervention differences at week 0 were tested using a Student’s independent samples t-test for normal distributed data and Mann-Whitney U test for non-normal distributed data. *Statistically significant (P <0.05). Abbreviations: BMI, body mass index; BP, blood pressure; CHO, carbohydrates; EGCG+RES, epigallocatechin-3-gallate and resveratrol; en, energy percentage of total energy intake; 2-h glucose, plasma glucose concentration 2 hours after oral glucose tolerance test; HbA1c, glycated hemoglobin A1c; MJ, megajoule; PLA, placebo; TNF-α, tumor necrosis factor-α.

**Supplemental Table 2.** Intervention effects of EGCG+RES on mitochondrial respiration in permeabilized skeletal muscle fibers (SkM) and fat oxidation.

|  | **Placebo** | | | **EGCG+RES** | | |  |
| --- | --- | --- | --- | --- | --- | --- | --- |
|  | **Week**  **0** | **Week 12** | ∆ | **Week**  **0** | **Week 12** | ∆ | **P-value** |
|  | **Mean** | **SD** | **%** | **Mean** | **SD** | **%** | **(time* treatment)** |
| *SkM-Ox* |  |  |  |  |  |  |  |
| ∆State 2, complex I (MG), pmol O_2_/mg muscle | 6.95 ± 3.96 | 4.22 ± 4.24* | -35.0 ± 87.8 | 3.37 ± 4.27 | 5.53 ± 3.04^#^ | 9.2 ± 13.1 | 0.006 |
| ∆State 3, complex I&II (MGS), pmol O_2_/mg muscle | 74.6 ± 15.39 | 72.48 ± 13.17* | -2.1 ± 9.0 | 60.66 ± 11.01 | 66.52 ±9.31* | 11.9 ± 18.6 | 0.010 |
| ∆Cytochrome c, pmol O_2_/mg muscle | 79.08 ± 17.10 | 76.37 ± 14.33 | -2.6 ± 10.3 | 63.29 ± 11.73 | 71.04 ± 12.31* | 14.4 ± 21.1 | 0.007 |
| ∆State 4o (oligomycin), pmol O_2_/mg muscle | 30.54 ± 10.46 | 27.7 ± 6.88 | -4.0 ± 22.6 | 24.83 ± 6.01 | 26.7 ± 8.91 | 9.4 ± 29.5 | 0.104 |
| ∆State uncoupled (FCCP), pmol O_2_/mg muscle | 101.67 ± 20.62 | 97.97 ± 18.41 | -2.4 ± 12.0 | 85.23 ± 13.91 | 91.47 ± 19.02^#^ | 7.2 ± 13.2 | 0.044 |
| *Fat oxidation* |  |  |  |  |  |  |  |
| Fasting, g/min | 0.08 ± 0.03 | 0.07 ± 0.04^#^ | -14.3 ± 47.6 | 0.07 ± 0.02 | 0.07 ± 0.03 | 10.5 ± 30.6 | 0.062 |
| Postprandial, AUC | 17.85 ± | 14.95 ± 5.18* | -16.5 ± 37.9 | 17.01 ± 5.75 | 17.75 ± 8.24 | 8.4 ± 24.0 | 0.034 |

Values are means ± SD. Data were analyzed by using repeated-measures ANOVA, with time (pre, post) and treatment (placebo, EGCG+RES) as factors. *P* < 0.05 was considered significant. In case of a significant time × treatment interaction, post hoc analyses with Bonferroni correction were applied to identify significant within-group effects. Within groups: **P* < 0.05, ^#^trend, *P* < 0.1. AUC, area under the curve; EGCG+RES, epigallocatechin-3-gallate and resveratrol; G, glutamate; M, malate; O_2_ skeletal muscle oxygen consumption; S, succinate.

**Supplemental Table 3.** Model estimates for EGCG+RES versus placebo intervention effects on relative taxa abundance on genus level.

|  | **Model** | **P-value** | **Q-value** |
| --- | --- | --- | --- |
| **Genus** | **estimate** | **(time*treatment*sex)** | **(time*treatment*sex)** |
| *Coprococcus 2* | -1.91 | 0.040 | 0.989 |
| *Fusicatenibacter* | 0.33 | 0.101 | 0.989 |
| *Ruminococcaceae UCG-005* | 0.94 | 0.116 | 0.989 |
| *Ruminococcaceae_NK4A214 group* | 0.86 | 0.133 | 0.989 |
| *Anaerostipes* | 0.29 | 0.140 | 0.989 |
| *Ruminococcus gnavus group* | -0.54 | 0.150 | 0.989 |
| *Streptococcus* | -0.91 | 0.157 | 0.989 |
| *Collinsella* | -0.78 | 0.184 | 0.989 |
| *Prevotella 9* | 0.88 | 0.216 | 0.989 |
| *Roseburia* | -0.37 | 0.230 | 0.989 |
| *Enterorhabdus* | -1.28 | 0.246 | 0.989 |
| *Lachnospiraceae FCS020 group* | 0.41 | 0.270 | 0.989 |
| *Ruminococcaceae UCG-003* | 1.19 | 0.284 | 0.989 |
| *Bilophila* | -0.74 | 0.296 | 0.989 |
| *Lachnospiraceae UCG-010* | -0.91 | 0.296 | 0.989 |
| *Phascolarctobacterium* | 0.48 | 0.367 | 0.989 |
| *Eubacterium eligens group* | 0.98 | 0.376 | 0.989 |
| *Ruminiclostridium 6* | -1.58 | 0.379 | 0.989 |
| *Veillonella* | -0.35 | 0.414 | 0.989 |
| *Eggerthella* | -1.33 | 0.423 | 0.989 |

Data represent the 20 genera with lowest P-values. No significant changes (Q < 0.2 (=False Discovery Rate (FDR) adjusted P-value)) in a generalized linear mixed model (Time*Treatment) corrected for sex.

**Supplemental Table 4.** Spearman correlations between pre-intervention relative abundance of microbial taxa at genus level and EGCG+RES induced changes mitochondrial respiration in permeabilized skeletal muscle fibers (SkM-Ox) in men and women.

|  | **Men (n = 8)** | | | **Women (n = 10)** | | |
| --- | --- | --- | --- | --- | --- | --- |
| **Genus** | **Spearman ρ** | **P-value** | **Q-value** | **Spearman ρ** | **P-value** | **Q-value** |
| ***∆State 2, complex I (MG)*** |  |  |  |  |  |  |
| *Dorea* | -1.00 | 0.000 | 0.000* | 0.43 | 0.397 | 0.840 |
| ***∆State 3, complex I&II (MGS)*** |  |  |  |  |  |  |
| *Barnsiella* | -0.94 | 0.005 | 0.170* | 0.58 | 0.228 | 0.726 |
| *Anaerotruncus* | -0.99 | 0.000 | 0.021* | -0.54 | 0.266 | 0.726 |
| *Ruminococcus 1* | -0.99 | 0.000 | 0.021* | -0.77 | 0.072 | 0.666 |
| *Ruminococcus 2* | -0.93 | 0.008 | 0.170* | -0.81 | 0.050 | 0.666 |
| *Subdoligranulum* | -0.93 | 0.008 | 0.170* | -0.14 | 0.787 | 0.888 |
| *Ruminococcaceae family genus* | -0.93 | 0.008 | 0.170* | -0.49 | 0.329 | 0.726 |
| ***∆Cytochrome c*** |  |  |  |  |  |  |
| *Barnsiella* | -1.00 | 0.000 | 0.000* | 0.41 | 0.425 | 0.703 |
| *uncultured bacterium_Ruminococcaceae* | -0.94 | 0.005 | 0.146* | -0.83 | 0.042 | 0.588 |
| *Coprococcus 1* | -0.93 | 0.008 | 0.146* | -0.71 | 0.111 | 0.607 |
| *Eubacterium ventriosum group* | -0.93 | 0.008 | 0.146* | -1.00 | 0.000 | 0.000* |
| *Anaerotruncus* | -0.93 | 0.008 | 0.146* | -0.60 | 0.208 | 0.611 |
| *Ruminococcaceae UCG-003* | -0.94 | 0.005 | 0.146* | 0.06 | 0.913 | 0.933 |
| Ruminococcus 1 | -0.93 | 0.008 | 0.146* | -0.83 | 0.042 | 0.588 |
| ***∆State 4o (oligomycin)*** |  |  |  |  |  |  |
| *Anaerotruncus* | -0.99 | 0.000 | 0.021* | 0.71 | 0.111 | 0.683 |
| *Ruminococcus 1* | -0.99 | 0.000 | 0.021* | 0.20 | 0.704 | 0.918 |
| ***∆State uncoupled (FCCP)*** |  |  |  |  |  |  |
| *Blautia* | 1.00 | 0.000 | 0.000* | -0.71 | 0.111 | 0.962 |

Spearman correlations between microbial taxa at week 0 and changes in SkM-Ox in the EGCG+RES group. Only significant correlations (Q < 0.2 (=False Discovery Rate (FDR) adjusted P-value)) and matched correlations for the other sex are shown. *Statistically significant (Q < 0.2). EGCG+RES, epigallocatechin-3-gallate and resveratrol; G, glutamate; M, malate; S, succinate***.***

**Supplemental Table 5.** Spearman correlations between pre-intervention relative abundance of microbial taxa at the genus level and EGCG+RES induced changes in fasting and postprandial fat oxidation in men and women. Only top 5 correlations based on (FDR-adjusted) P-value are shown.

|  | **Spearman ρ** | **P-value** | **Q-value** |
| --- | --- | --- | --- |
| **Men (n = 8)** |  |  |  |
| ***Fasting fat oxidation (Δ 12 wk)*** |  |  |  |
| *[Eubacterium] coprostanoligenes group* | -0.88 | 0.004 | 0.547 |
| *Actinomyces* | 0.63 | 0.096 | 0.705 |
| *Bifidobacterium* | 0.69 | 0.058 | 0.705 |
| *Butyricimonas* | 0.59 | 0.122 | 0.705 |
| Bacillus | 0.58 | 0.134 | 0.705 |
| ***Postprandial fat oxidation (Δ 12 wk)*** |  |  |  |
| *Dorea* | -0.87 | 0.005 | 0.320 |
| *Lachnospiraceae UCG-010* | -0.87 | 0.005 | 0.321 |
| *Clostridium sensu stricto 1* | -0.83 | 0.010 | 0.468 |
| *Bacteroides* | 0.76 | 0.028 | 0.966 |
| *Methanobrevibacter* | -0.05 | 0.912 | 0.976 |
| **Women (n = 10)** |  |  |  |
| ***Fasting fat oxidation (Δ 12 wk)*** |  |  |  |
| *Actinomyces* | 0.52 | 0.122 | 0.658 |
| *Slackia* | 0.68 | 0.031 | 0.658 |
| *Uncultured Porphyromonadaceae bacterium* | 0.52 | 0.122 | 0.658 |
| *Prevotellaceae UCG-001* | 0.52 | 0.122 | 0.658 |
| *Gemella* | -0.52 | 0.122 | 0.658 |
| ***Postprandial fat oxidation (Δ 12 wk)*** |  |  |  |
| *Actinomyces* | 0.27 | 0.476 | 0.919 |
| *Bifidobacterium* | 0.17 | 0.668 | 0.919 |
| *Adlercreutzia* | -0.42 | 0.256 | 0.919 |
| *Collinsella* | -0.46 | 0.213 | 0.919 |
| *Enterorhabdus* | -0.22 | 0.573 | 0.919 |

Data represent the 5 genera with lowest P-values for correlations with EGCG+RES induced changes in fasting and postprandial fat oxidation. Fat oxidation was determined by indirect calorimetry during fasting conditions and for 4 hours after ingesting a liquid high-fat mixed-meal. No significant correlations (Q < 0.2 (=False Discovery Rate (FDR) adjusted P-value)) were found. EGCG+RES, epigallocatechin-3-gallate and resveratrol.

**Supplemental Table 6.** Spearman correlations between EGCG+RES induced changes in relative abundance of microbial taxa at genus level and EGCG+RES induced changes mitochondrial respiration in permeabilized skeletal muscle fibers (SkM-Ox) in men.

|  | **Men** | | |
| --- | --- | --- | --- |
| ***Genus*** | **Spearman ρ** | **P-value** | **Q-value** |
| **∆State 2, complex I (MG)** |  |  |  |
| *∆Ruminococcaceae UCG 004* | -0.93 | 0.008 | 0.093 |
| **∆State 3, complex I&II (MGS)** |  |  |  |
| *∆Ruminococcus 1* | 0.99 | 0.000 | 0.006 |
| *∆Ruminococcus 2* | 0.99 | 0.000 | 0.006 |
| *∆Anaerofilum* | 0.94 | 0.005 | 0.077 |
| *∆Erysipelotrichaceae D 5 uncultured* | 0.93 | 0.008 | 0.107 |
| *∆Lachnospiraceae Ambiguous taxa* | 0.90 | 0.015 | 0.180 |
| *∆Marvinbryantia* | -0.90 | 0.015 | 0.180 |
| **∆Cytochrome c** |  |  |  |
| *∆Erysipelotrichaceae D 5 uncultured* | 0.99 | 0.000 | 0.007 |
| *∆Coprococcus 1* | 0.94 | 0.005 | 0.087 |
| *∆Ruminococcus 1* | 0.93 | 0.008 | 0.116 |
| *∆Ruminococcus 2* | 0.93 | 0.008 | 0.116 |
| **∆State 4o (oligomycin)** |  |  |  |
| *∆Lachnospiraceae Ambiguous taxa* | 0.99 | 0.000 | 0.005 |
| *∆Marvinbryantia* | -0.99 | 0.000 | 0.005 |
| *∆Ruminococcus 1* | 0.99 | 0.000 | 0.005 |
| *∆Ruminococcus 2* | 0.99 | 0.000 | 0.005 |
| *∆Coprococcus 1* | 0.94 | 0.005 | 0.058 |
| *∆Eubacterium ventriosum group* | 0.94 | 0.005 | 0.058 |
| *∆Anaerofilum* | 0.94 | 0.005 | 0.058 |
| *∆Ruminiclostridium 5* | 0.94 | 0.005 | 0.058 |
| *∆Ruminococcaceae UCG 005* | 0.93 | 0.008 | 0.077 |
| *∆Erysipelotrichaceae D 5 uncultured* | 0.93 | 0.008 | 0.077 |
| *∆Christensenellaceae R 7 group* | 0.89 | 0.019 | 0.171 |
| *∆Ruminococcaceae NK4A214 group* | 0.88 | 0.021 | 0.178 |
| **∆State uncoupled (FCCP)** |  |  |  |
| *∆Lachnospiraceae* | 0.94 | 0.005 | 0.092 |
| *∆Ruminococcaceae UCG 003* | 0.94 | 0.005 | 0.092 |

Spearman correlations between changes in microbial taxa and changes in SkM-Ox in men within the EGCG+RES group. Only significant correlations (Q < 0.2 (=False Discovery Rate (FDR) adjusted P-value)) are shown. *Statistically significant (Q < 0.2). EGCG+RES, epigallocatechin-3-gallate and resveratrol; G, glutamate; M, malate; S, succinate.

**Supplemental Table 7.** Spearman correlations between EGCG+RES induced changes in relative abundance of microbial taxa at genus level and EGCG+RES induced changes mitochondrial respiration in permeabilized skeletal muscle fibers (SkM-Ox) in women.

|  | **Women** | | |
| --- | --- | --- | --- |
| ***Genus*** | **Spearman ρ** | **P-value** | **Q-value** |
| **∆State 2, complex I (MG)** |  |  |  |
| *Ruminococcus 1* | 0.943 | 0.00 | 0.055 |
| **∆State 3, complex I&II (MGS)** |  |  |  |
| *Enterobacteriaceae* | 0.986 | 0.00 | 0.007 |
| *Lachnospiraceae NK4A136 group* | -0.943 | 0.00 | 0.079 |
| *Roseburia* | -0.943 | 0.00 | 0.079 |
| **∆Cytochrome c** |  |  |  |
| Enterobacteriaceae | 0.928 | 0.01 | 0.155 |
| **∆State 4o (oligomycin)** |  |  |  |
| *Ruminococcus torques group* | 0.943 | 0.00 | 0.079 |
| **∆State uncoupled (FCCP)** |  |  |  |
| *Peptostreptococcaceae* | 0.943 | 0.00 | 0.079 |

Spearman correlations between changes in microbial taxa and changes in SkM-Ox in women within the EGCG+RES group. Only significant correlations (Q < 0.2 (=False Discovery Rate (FDR) adjusted P-value)) are shown. *Statistically significant (Q < 0.2). EGCG+RES, epigallocatechin-3-gallate and resveratrol; G, glutamate; M, malate; S, succinate.
